# Transcriptional and Non-Transcriptional Functions of PPARβ/δ in Non-Small Cell Lung Cancer

**DOI:** 10.1371/journal.pone.0046009

**Published:** 2012-09-25

**Authors:** Davide Genini, Ramon Garcia-Escudero, Giuseppina M. Carbone, Carlo V. Catapano

**Affiliations:** 1 Institute of Oncology Research (IOR) and Oncology Institute of Southern Switzerland (IOSI), Bellinzona, Switzerland; 2 Molecular Oncology Unit, Centro de Investigaciones Energéticas, Medioambientales y Tecnológicas (CIEMAT), Madrid, Spain; University Magna Graecia, Italy

## Abstract

Peroxisome proliferator-activated receptor β/δ (PPARβ/δ) is a nuclear receptor involved in regulation of lipid and glucose metabolism, wound healing and inflammation. PPARβ/δ has been associated also with cancer. Here we investigated the expression of PPARβ/δ and components of the prostaglandin biosynthetic pathway in non-small cell lung cancer (NSCLC). We found increased expression of PPARβ/δ, Cox-2, cPLA_2_, PGES and VEGF in human NSCLC compared to normal lung. In NSCLC cell lines PPARβ/δ activation increased proliferation and survival, while PPARβ/δ knock-down reduced viability and increased apoptosis. PPARβ/δ agonists induced Cox-2 and VEGF transcription, suggesting the existence of feed-forward loops promoting cell survival, inflammation and angiogenesis. These effects were seen only in high PPARβ/δ expressing cells, while low expressing cells were less or not affected. The effects were also abolished by PPARβ/δ knock-down or incubation with a PPARβ/δ antagonist. Induction of VEGF was due to both binding of PPARβ/δ to the VEGF promoter and PI3K activation through a non-genomic mechanism. We found that PPARβ/δ interacted with the PI3K regulatory subunit p85α leading to PI3K activation and Akt phosphorylation. Collectively, these data indicate that PPARβ/δ might be a central element in lung carcinogenesis controlling multiple pathways and representing a potential target for NSCLC treatment.

## Introduction

Peroxisome proliferator-activated receptors (PPARs) are nuclear hormone receptors (NHRs) activated by lipophilic ligands, including long chain fatty acids and prostaglandins [1]. PPARs form heterodimers with the retinoid X receptor (RXR) and bind to specific elements in gene promoters. PPARs are involved in metabolic and developmental processes. PPARβ/δ has an important role in lipid and glucose metabolism and is an attractive therapeutic target for metabolic and degenerative disorders [1]. PPARβ/δ is also implicated in inflammation, wound healing, cell growth and differentiation. PPARβ/δ is over-expressed in human cancers and may be important in tumor initiation and progression [1]. In support of a pro-tumorigenic function, PPARβ/δ ligands promoted cancer cell survival in vitro [2], [3], [4] and tumor growth in mice [5], [6], [7]. Conversely, genetic knock-out of PPARβ/δ in colon cancer cells decreased tumor growth in mice [8]. Other data, however, using agonists and genetic knock-out in cellular and mouse models contradict this tumor promoting function [9]. Knock-out of PPARβ/δ in colon cancer models was reported to promote tumor formation in mice, while agonists reduced cell proliferation in vitro and tumor growth in mice [10], [11], [12]. Various factors could affect the response to ligand activation, over-expression and knock-out of PPARβ/δ. We reported previously that expression and activity of PPARβ/δ varied considerably in human NSCLC cell lines and PPARβ/δ protein level depended on the ligands ability to protect from proteosomal degradation [13]. The basal level of the receptor and this post-transcriptional regulatory step could account in part for the variable responses to PPARβ/δ agonists in different experimental conditions [14].

During wound healing and inflammation PPARβ/δ function is associated with induction of cyclooxygenase-2 (Cox-2) [1]. Cox-2 transforms arachidonic acid released by phospholipase A_2_ (cPLA_2_) into PGH_2_ [15]. PGH_2_ is then transformed into prostaglandins, like prostaglandin E_2_ (PGE_2_) and prostaglandin I_2_ (PGI_2_), endowed of complex biological activities. Arachidonic acid and PGI_2_ act as PPARβ/δ agonists [2], while PGE_2_ enhances the activity of PPARβ/δ without directly binding to the receptor [6]. Non steroidal anti-inflammatory drugs (NSAIDs) and Cox-2 inhibitors affect PPARβ/δ by preventing production of prostaglandins [3]. On the other hand, increased expression of PPARβ/δ has been reported to protect cancer cells from the antiproliferative and pro-apoptotic effects of NSAIDs and Cox-2 inhibitors [3]. Cox-2 is over-expressed in pre-malignant and malignant lesions, including lung cancers [16], and has an important role in tumor associated inflammation and angiogenesis [15]. Furthermore, PPARβ/δ and Cox-2 can impact on the production of pro-inflammatory and pro-angiogenic factors in tumors, like vascular endothelial growth factor (VEGF) [17], [18]. Thus, current evidence places PPARβ/δ along with Cox-2 and prostaglandin synthases within signaling pathways that might control proliferation and survival of cancer cells and their interaction with the tumor microenvironment.

Lung cancer is a leading cause of cancer death worldwide [19]. Non–small cell lung cancer (NSCLC) represents about 85% of all lung cancers. NSCLC is often diagnosed at an advanced stage and has a very poor prognosis. A better understanding of the factors involved in the origin and progression of NSCLC could lead to improvement in the treatment and prevention. In this study we investigated whether and how PPARβ/δ could contribute to the pathogenesis of NSCLC. We found that PPARβ/δ was frequently up-regulated in NSCLC compared to normal lung. PPARβ/δ over-expression was generally associated with increased expression of cPLA_2_, Cox-2, PGES and VEGF. We examined the consequences of PPARβ/δ activation on cell proliferation and survival and on expression of Cox-2 and VEGF in NSCLC cell lines. We found evidence consistent with a pro-tumorigenic role of PPARβ/δ in NSCLC. Additionally, we found that PPARβ/δ agonists led to induction of VEGF also through a parallel non-transcriptional mechanism linked to PI3K/Akt activation. Collectively, these data indicate that PPARβ/δ might be a central element in lung carcinogenesis controlling multiple processes and pathways and thus representing a potential target for development of novel strategies for lung cancer treatment.

## Materials and Methods

### Cell Lines

Human lung carcinoma cell lines H358, H441, H23 and A549 were purchased from American Type Culture Collection (LGC Promochem, Molsheim, F) and were maintained in RPMI supplemented with 10% FBS. Cells were grown in phenol red-free RPMI supplemented with 5% charcoal-stripped serum (HyClone, Logan, UT, USA) prior to incubation with PPAR ligands.

### Chemicals

GW501516, LY294002 and ciglitazone were purchased from Alexis (Lausanne, Switzerland). cPGI_2_ was purchased from Biomol (Plymouth Meeting, PA). L165041 and NS398 were obtained from Sigma (Buchs, CH). Wortmannin was purchased from Calbiochem (Merk Biosciences, Nottingham, UK). The PPARβ/δ antagonist GSK0660 was supplied by Dr. A. Billin (GlaxoSmithKline, USA). All compounds were dissolved in DMSO.

### Patient Samples

Lung cancer specimens (squamous cell carcinomas and adenocarcinomas, stage IA to IIIA) and adjacent normal lung tissue samples were from the Medical University of South Carolina (Charleston, SC, USA) and were obtained at the time of surgery with patient informed consent. Tissue samples were snap-frozen and stored in liquid nitrogen. RNA was isolated using RNA STAT-60. RT-PCR was performed using 100 ng of total RNA and 0.4 µM of primers with SuperScript One-Step RT-PCR (Invitrogen). PCR products were analyzed by agarose gel electrophoresis, visualized using the AlphaImager (AlphaInnotech) and quantified by densitometric analysis using the AlphaImager software. Results were presented as ratio between the band intensity in paired tumor and normal samples normalized to the reference gene β-actin. Pearson correlation analysis was performed on the normalized gene expression levels. Genome-wide transcriptome datasets from human lung cancer and normal lung tissue samples from four different studies (PMID: 18992152, 11707590, 20421987, 18641660) were used to examine correlations between PPARβ/δ and putative target genes. Normalized gene expression values for each transcript were downloaded and Pearson correlation coefficient and the corresponding p-value with respect to PPARβ/δ were calculated for the samples in each dataset.

### Luciferase Assay

The PPARβ/δ responsive reporter (DRE) was provided by B. Vogelstein [3]. Cells were transfected with DRE or basic pGL3 luciferase reporter along with pRL-SV40 control plasmid using Lipofectamine. Cells were grown in RPMI medium supplemented with 5% charcoal-stripped serum for 24 h. Luciferase activity was measured using the Dual Luciferase kit (Promega) as described [20].

### Cell Proliferation and Viability

Cells were plated in 96-well plates in phenol red-free RPMI supplemented with 5% charcoal-stripped serum. After 24 h cells were treated with ligands or DMSO (0.1%) in 0.1% charcoal-stripped serum. Number of viable cells was determined using MTT after 72 h [20]. All assays were performed in triplicate and repeated in at least three independent experiments. For cell cycle analysis cells were grown in 0.1% charcoal-stripped serum and harvested after 24 h of incubation with ligands or DMSO. Cells were then stained with propidium iodide and analyzed by flow cytometry as described [20].

### RNA Interference

For knock-down experiments, cells were seeded at low concentration (30–50% confluence) and transfected with 10 nM siRNA (Ambion, Huntingdon, UK) directed to PPARβ/δ (siPPARβ/δ) and firefly luciferase (siGL3) using Interferin according to the manufacturer’s suggested protocol (Polyplus). Transfection of siRNA was repeated every three days for three times. Cell viability was determined using MTT after 72 h of growth in 0.1% of charcoal-stripped serum from the last transfection. Apoptosis was similarly assessed after 72 h by Annexin V-FITC and flow cytometry as described [20].

### RNA Isolation and RT-PCR

Cells (1×10^6^ cells) were plated in 60-mm dishes, grown in serum and phenol red free RPMI for 24 h, and then incubated with various compounds for 18 h. RNA was isolated using Trizol (Invitrogen) and RNeasy MiniKit (Qiagen). RT-PCR was performed under non-saturating conditions using the SuperScript™ III One-Step RT-PCR System (Invitrogen) and gene-specific primers (Table S1) [20]. PCR products were separated on 2% agarose gels, stained with GelRed (Biotium, Basel, CH) and quantified using the AlphaImager as described above.

### Immunoblotting

Cells were lysed as described [13]. Lysates were centrifuged at 14,000× g for 10 minutes to remove any debris and protein concentration was determined. Proteins were loaded on 10–12% polyacrylamide gels and analyzed by immunoblotting. Procaspase-3 (302), PDK1, PTEN, Akt, and phospho-Akt (Ser 473) antibodies were purchased from Cell Signaling Technology (Danvers, MA). Cox-2 (E-29) and PPARβ/δ (H-74) antibodies were obtained from St Cruz (Heidelberg, Germany). Tubulin (Ab-1) antibody was purchased from Oncogene (Merk Biosciences, Nottingham, UK).

### Chromatin Immunoprecipitation (ChIP)

Cells were grown to confluence in 75-cm^2^ flasks, starved, and incubated with GW501516 or vehicle for 18 h. ChIP was performed as described [21] with 4 µg anti-PPARβ/δ antibody or anti-IgG as a negative control. PCR was done with primers spanning the regions from −527 to −298 and from −1338 to −1123 of the VEGF promoter in the presence of Betaine and DMSO using AmpliTaq Gold (Applied Biosystem, Foster City, CA). Densitometric analysis was performed using the AlphaImager software.

### His-tagged PPARβ/δ Pull-down and Immunoprecipitation

The His-PPARβ/δ expression vector was described previously [13]. The His-tagged N- and C- terminal truncated constructs of PPARβ/δ were generated by site directed mutagenesis. Cells were transfected with Lipofectamine, grown to confluence and incubated with and without PPARβ/δ ligands. Cells were lysed in RIPA buffer for 30 minutes on ice and subject to pull-down with His-select nickel affinity gel (Sigma). Proteins were eluted with Laemmli buffer. Equivalent aliquots of lysates, flow-through and eluates were loaded on gels and analyzed by immunoblotting. Immunoprecipitation (IP) was performed using anti-p85α serum (a gift of M. Thelen, IRB, Bellinzona, CH) and A/G sepharose beads (Thermo Scientifics). His-PPARβ/δ was detected with an anti-His antibody (Sigma).

## Results

### Expression of PPARβ/δ in Non-small Cell Lung Cancer

**Figure 1:**
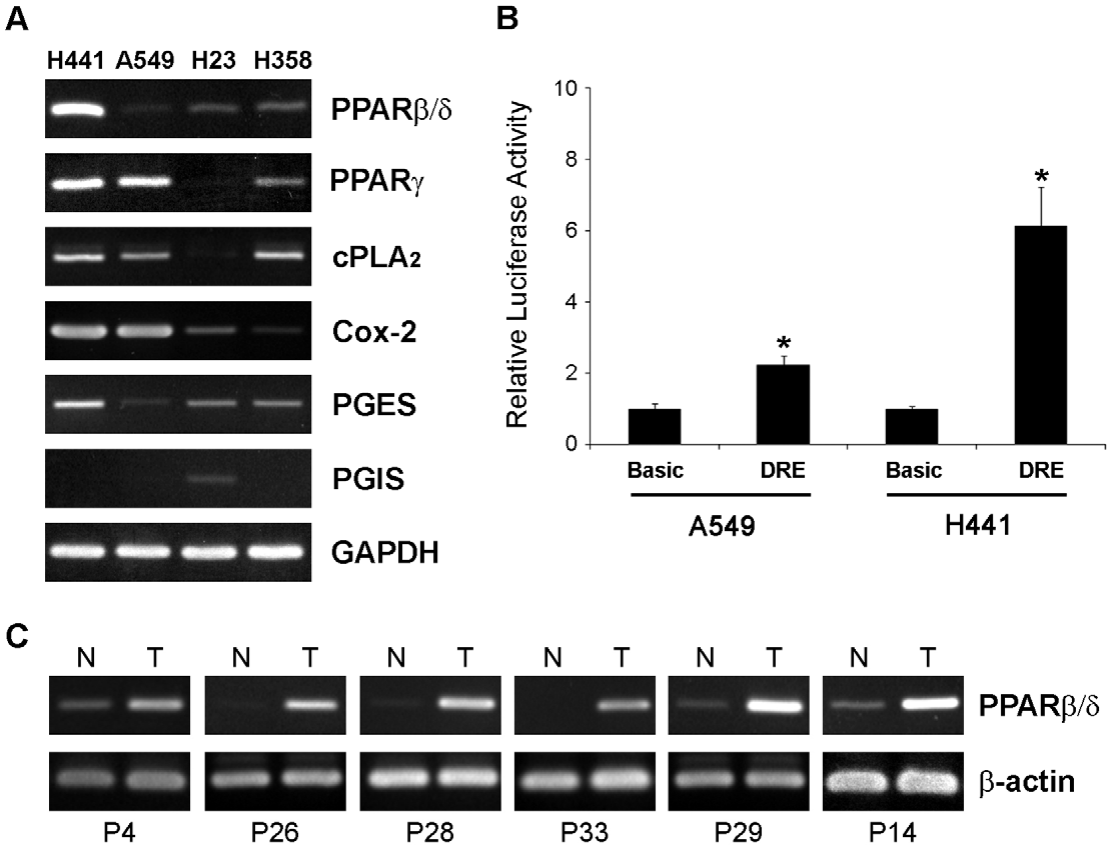
Expression of PPARβ/δ in non-small cell lung cancer cell lines and tumor samples. (**A**) RNA isolated from the indicated cell lines was amplified by RT-PCR to assess the level of PPARβ/δ, PPARγ, cPLA_2_, Cox-2, PGIS, and PGES RNA. GAPDH was used as a reference gene. (**B**) H441 and A549 cells were transfected with a PPARβ/δ responsive luciferase reporter (DRE) or basic pGL3 luciferase reporter (Basic). Luciferase activity was assessed after 24 h. * P<0.01. (**C**) RNA isolated from lung tumors and adjacent normal lung tissue was analyzed by RT-PCR with primers specific for PPARβ/δ and β-actin.

We assessed the level of PPARβ/δ along with PPARγ, cPLA_2_, Cox-2, PGES and PGIS in NSCLC cell lines. Expression of these genes varied considerably among the cell lines (Fig. 1A). H441 cells had high levels of PPARβ/δ, Cox-2, cPLA_2_, PGES and PPARγ. H358 and H23 cells had intermediate levels of PPARβ/δ and low/moderate expression of Cox-2, PGES, cPLA_2_ and PPARγ. Interestingly, A549 cells, which have been used in many studies to test the effects of PPARβ/δ agonists, had the lowest level of PPARβ/δ and PGES, while had relatively high expression of PPARγ and Cox-2. The difference in PPARβ/δ level between H441 and A549 cells was confirmed using a selective PPARβ/δ responsive luciferase reporter [3] (Fig. 1B) and was consistent with previous data from our group on protein level and response to ligand activation in the two cell lines [13]. Interestingly, most of the NSCLC cell lines did not express PGIS, with the exception of H23 cells in which a low level of PGIS mRNA was detected. This suggested that PGI_2_ is unlikely to be produced endogenously in most NSCLC cells.

**Figure 2:**
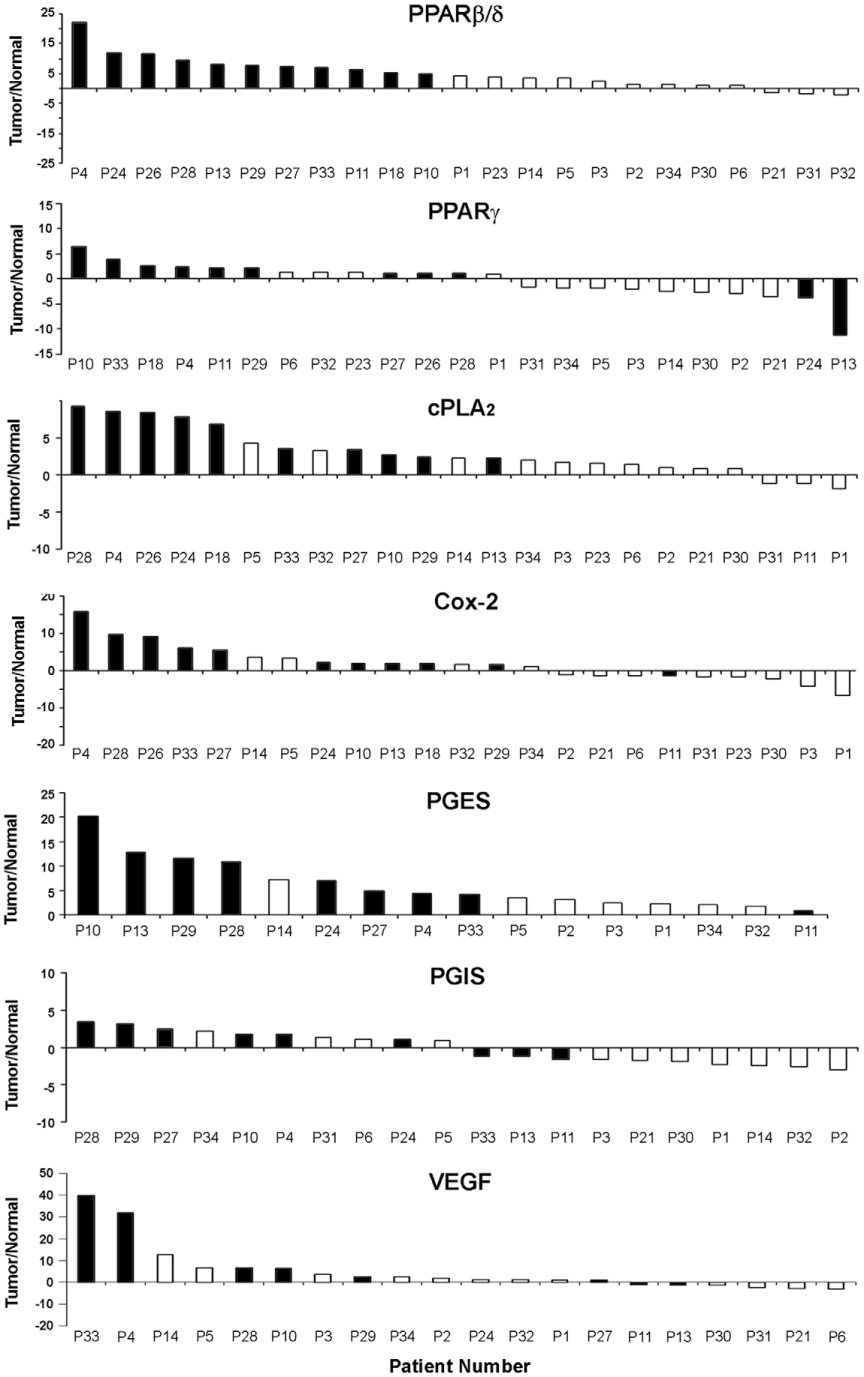
Expression of PPARβ/δ, PPARγ, cPLA_2_, Cox-2, PGES, PGIS, and VEGF in non-small cell lung cancers. RNA was extracted from tumors and adjacent normal lung tissue from patients with non-small cell lung cancer and examined by RT-PCR. Data represent the ratio of gene expression in tumors relative to the paired normal tissue based on densitometric analysis and normalized to β-actin used as reference gene. Black bars mark the tumors with highest expression of PPARβ/δ (T/N ratio ≥4).

Next, we examined the expression of the same genes in normal lung and tumor tissue samples from patients with NSCLC (Figure S1). We found increased PPARβ/δ mRNA in many tumors compared to the paired normal lung samples (Fig. 1C). To compare the pattern of expression in the entire sample set, mRNA level of each gene was determined by densitometric analysis, normalized to β-actin and presented as ratio of the level in each pair of tumor/normal matched samples (Fig. 2). PPARβ/δ mRNA was markedly up-regulated (T/N ratio ≥4) in about 50% of tumors. This is in line with previous reports of over-expression of this NHR in many human cancers, including NSCLC [1], [22]. Cox-2, cPLA_2_ and PGES were also up-regulated in most tumor samples (Fig. 2). Up-regulation of these genes was particularly evident in tumors with PPARβ/δ over-expression. Notably, PGIS was detected in normal lung and changed only slightly in a small fraction of cases. PPARγ was moderately increased in some tumors but more frequently down-regulated, consistent with the putative tumor suppressor role attributed to this NHR. VEGF, which is a putative target of PPARβ/δ and Cox-2, was also up-regulated in many tumors with PPARβ/δ over-expression (Fig. ). Notably, the level of PPARβ/δ correlated significantly with the expression of Cox-2 (Pearson coeff. 0.76; p-value, 2.91E−05), cPLA_2_ (Pearson coeff. 0.69; p-value 2.72E−04) and VEGF (Pearson coeff. 0.54; p-value 0.018). To provide further support to the link between PPARβ/δ, VEGF and Cox-2 we examined the level of these genes in publicly available gene expression datasets from normal and lung cancer tissue samples. We found significant correlations of PPARβ/δ with VEGF and Cox-2 mRNA expression in multiple datasets (Table S2). Taken together, these data indicate frequent and concomitant up-regulation of PPARβ/δ, VEGF and components of the Cox-2/prostaglandin synthetic pathway in a subset of NSCLC and provide support to the hypothesis that activation of these pathways may play a role in lung carcinogenesis.

### PPARβ/δ Promotes Proliferation and Survival of Non-small Cell Lung Cancer Cells

**Figure 3:**
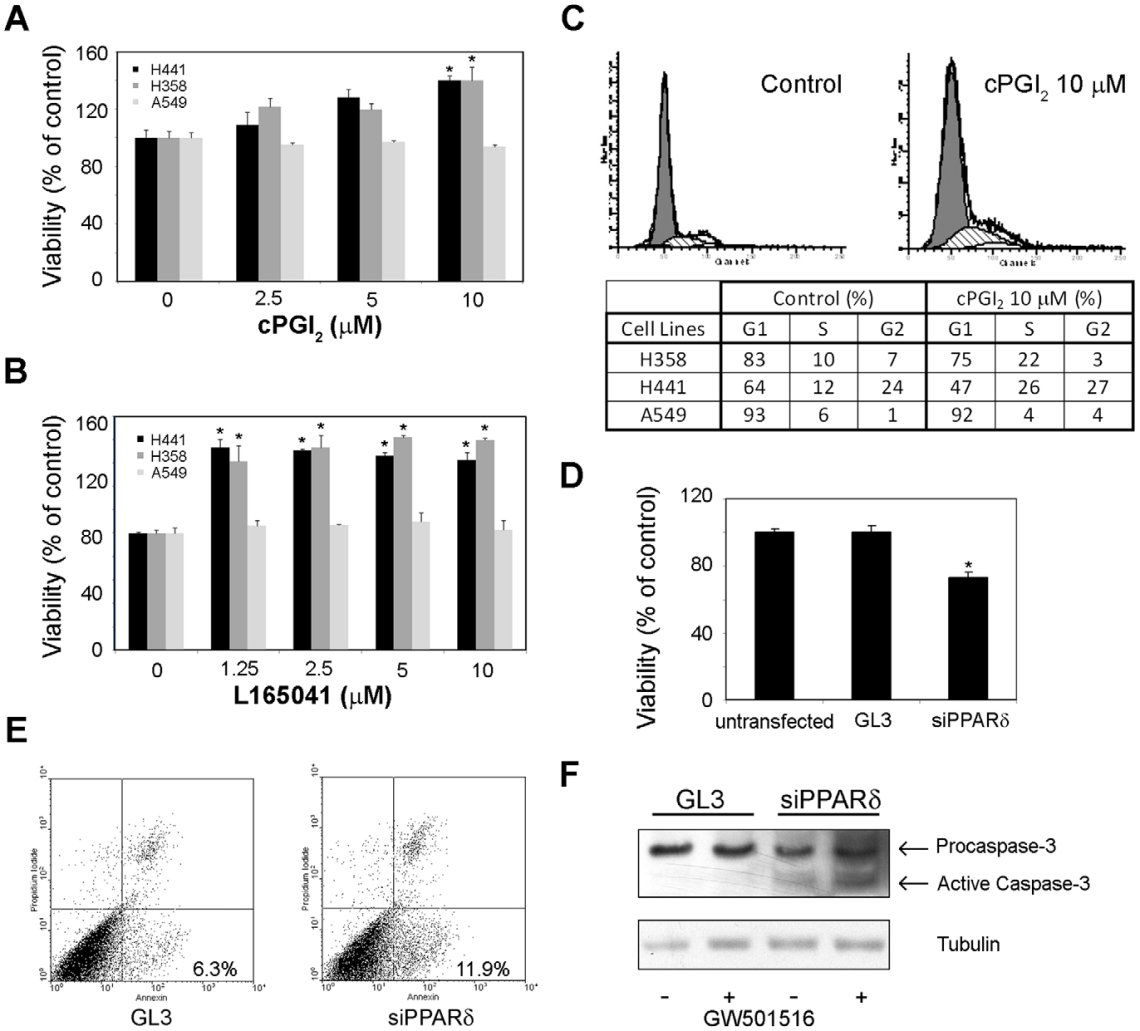
PPARβ/δ activation affects growth and survival of non-small cell lung cancer cells. (**A**) H441, H358 and A549 cells were incubated with cPGI_2_ in serum-free medium and cell viability was assessed after 72 h with MTT assay. *P<0.01 relative to control cells. (**B**) Cells were incubated with L165041 as above. *P<0.01 relative to control cells. (**C**) Cells were incubated with cPGI_2_ (10 µM) for 24 h and analyzed by flow cytometry. *Top panel*, representative flow cytometric profile of H358 cells incubated with and without cPGI_2_. *Bottom panel*, cell cycle distribution in cells after 24 h incubation with and without cPGI_2_. The increase in S phase cells in H441 and H358 cells determined in triplicate experiments was statistically significant (P<0.01). (**D**) H441 cells were transfected with siRNA for PPARβ/δ and GL3 and cell viability was determined after 72 h with MTT. *P<0.01 relative to control cells. (**E**) H358 cells transfected with PPARβ/δ siRNA and GL3 were stained with annexin V and propidium iodide and analyzed by flow cytometry. The percentage of annexin V positive cells (apoptotic cells) is indicated in each panel. P<0.05. (**F**) H358 cells were transfected with siRNA for PPARβ/δ and GL3, lysed and analyzed by immunoblotting with a caspase-3 antibody. *P<0.01.

Deregulated expression of PPARβ/δ can favor proliferation and survival of cancer cells. However, studies done in various cell models, including lung cancer cell lines failed to provide consistent results [9], [23]. The data described above suggest that the context in which some studies were done was quite heterogeneous and could affect the diverse cellular responses to PPARβ/δ activation. We found that PPARβ/δ agonists increased proliferation and promoted survival of NSCLC cells. Activation of PPARβ/δ by cPGI_2_ in low serum medium increased cell viability and proliferation (Fig. 3A). Similar effects were seen with another PPARβ/δ agonist, L165041 (Fig. 3B). Consistently, cell cycle analysis showed an increase of S-phase cells after treatment with cPGI_2_ in low serum medium while G1 phase cells decreased (Fig. 3C). Notably, all these effects were evident in cells with high expression of PPARβ/δ (e.g., H441 and H358), while there was no or minimal effect on growth and cell cycle in A549 cells with low level of the receptor (Fig. 3A–C). These pro-growth and survival effects were specific for PPARβ/δ agonists as incubation with the PPARγ agonist ciglitazone inhibited cell growth (Figure S2). In addition, high expression of PPARβ/δ was associated with reduced sensitivity to NSAIDs and Cox-2 inhibitors, like sulindac sulfide, sulindac sulfone and NS398, in H441 cells compared to A549 cells with low PPARβ/δ expression (Figure S2). In support of a pro-survival function, knock-down of PPARβ/δ using small interfering RNA (siRNA) affected cell viability (Fig. 3D). PPARβ/δ knock-down increased also the number of Annexin V positive apoptotic cells (Fig. 3E) and induced caspase-3 activation, a known marker of apoptotic cell death (Fig. 3F). Taken together, these data indicate that activation of PPARβ/δ promotes survival and proliferation of NSCLC cells that express high levels of the receptor.

### PPARβ/δ Activation Induces Cox-2 and VEGF Expression

**Figure 4:**
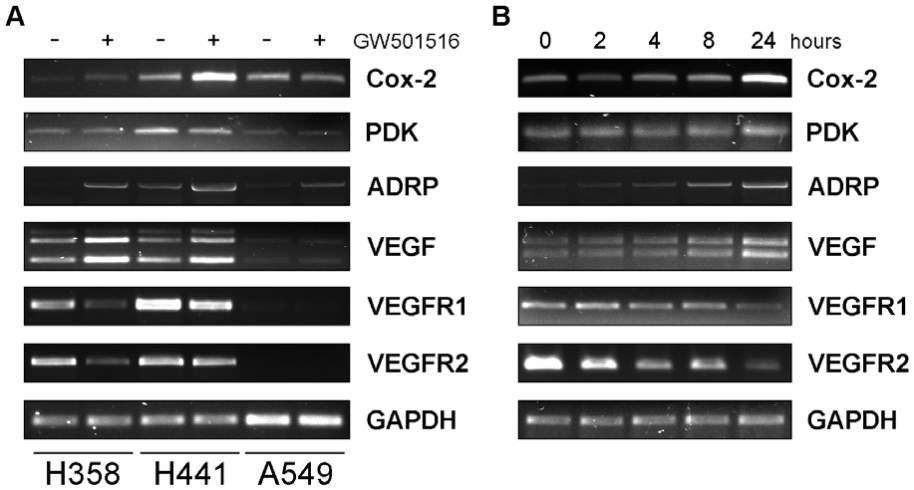
PPARβ/δ activation modulates gene expression. (**A**) H358, H441 and A549 cells were grown to confluence, starved for 24 h and then treated with GW501516 (5 µM) for 18 h. Total RNA was isolated and examined by RT-PCR. (**B**) H358 cells were incubated with GW501516 for the indicated times prior to RNA extraction and analysis.

Cox-2 and PPARβ/δ can functionally interact and reciprocally regulate each other. The concomitant up-regulation of PPARβ/δ and components of the Cox-2/prostaglandin synthetic pathway in NSCLC tissues and cell lines further supported this link and induced us to test whether PPARβ/δ could affect Cox-2 expression in NSCLC cells. We observed an increase of Cox-2 mRNA upon treatment of NSCLC cells with the PPARβ/δ ligand GW501516 (Fig. 4A). Notably, Cox-2 mRNA did not increase in A549 cells suggesting that the effect depended on the level of endogenous PPARβ/δ. GW501516 induced also transcription of the adipose differentiation-related protein (ADRP) gene, which is a known target of PPARβ/δ, in H358 and H441 cells and only to a minor extent in A549 cells (Fig. 4A). On the contrary, PDK, a PPARβ/δ target gene reported in other studies [24], was not affected (Fig. 4A). A time-course analysis showed that the changes in Cox-2 and ADRP mRNA level were evident within 4–8 h from the addition of the ligand and increased further at 24 h (Fig. 4B).

PPARβ/δ and Cox-2 could constitute a feed-forward regulatory loop sustaining cell survival and proliferation. In addition, PPARβ/δ was identified as a key component of the angiogenic switch during tumor progression [25] and VEGF, which is the major mediator of angiogenesis, was identified as a target of PPARβ/δ [18]. Analysis of VEGF expression in NSCLC and normal lung samples showed increased expression of VEGF and high correlation with PPARβ/δ in lung cancers (Fig. 2 and Table S2), consistent with the idea that PPARβ/δ could regulate VEGF expression. To test the effect of PPARβ/δ on VEGF expression, we treated NSCLC cells with GW501516. VEGF mRNA increased in H358 and H441 cells incubated with GW501516, while did not change in A549 cells (Fig. 4A). As seen for Cox-2 and ADRP, VEGF mRNA increased within 4–8 h and increased further after 24 h (Fig. 4B). To our surprise, expression of the VEGF receptors VEGFR1 and VEGFR2 was reduced upon treatment with GW501516 (Fig. 4A–B).

### PPARβ/δ is Directly Involved in Regulation of VEGF Transcription

**Figure 5:**
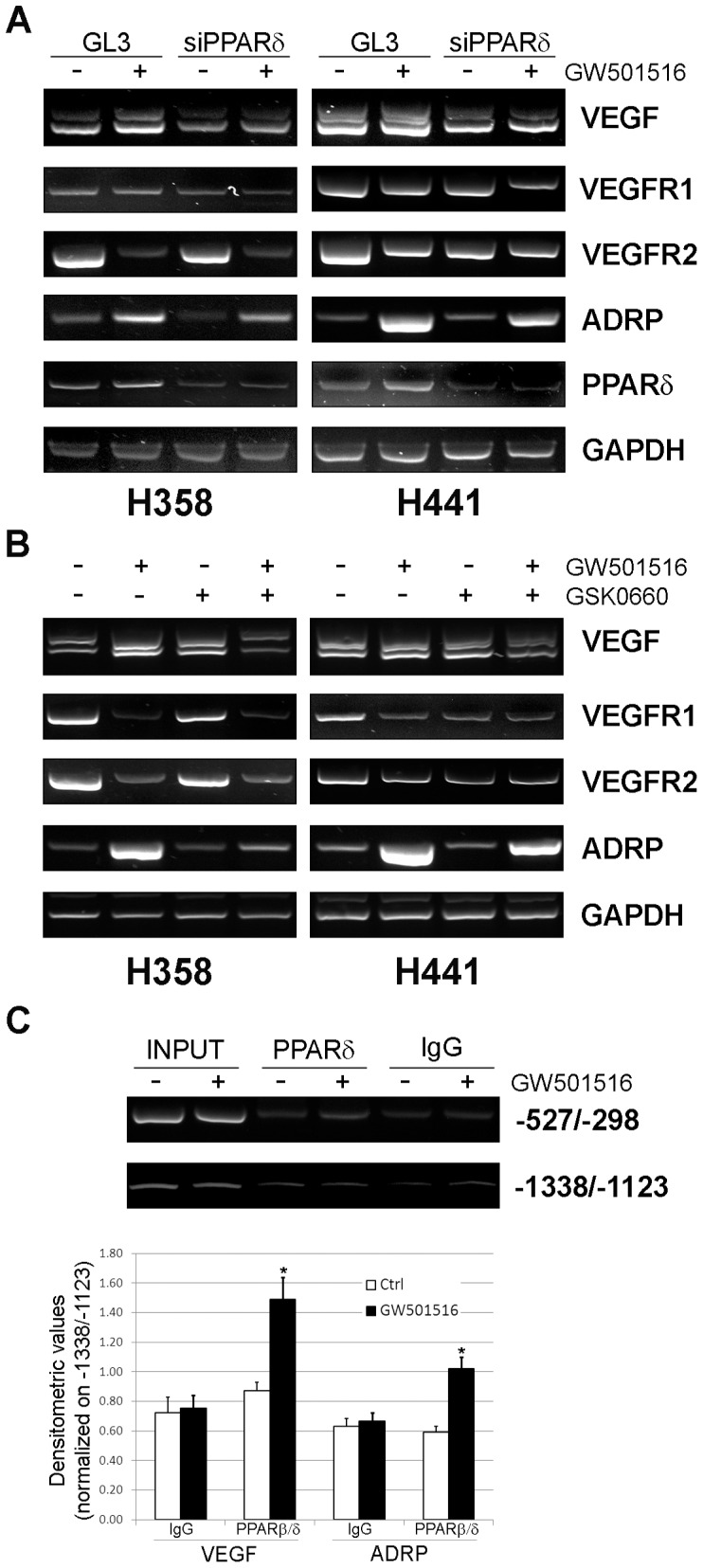
PPARβ/δ knock-down and antagonist prevent VEGF induction. (**A**) H358 and H441 cells were transfected with PPARβ/δ siRNA and control GL3 and then treated with GW501516 (5 µM) for 18 h. Gene levels were determined by RT-PCR. (**B**) Cells were incubated for 2 h with GSK0660 (10 µM) and then with GW501516 (5 µM) for 18 h prior analysis by RT-PCR. (**C**) H358 cells were treated with GW501516 for 18 h and processed for chromatin immunoprecipitation using anti-PPARβ/δ and anti-IgG antibodies. The region of the VEGF promoter containing a PPRE (−527/−298) and a non-targeted region (−1338/−1123) were PCR amplified. The PPRE containing region of the ADRP promoter was amplified as positive control. *Top panel*, representative gel scan. *Bottom panel*, densitometric quantification of three independent experiments in H441 cells. *P<0.01.

Induction of VEGF expression by PPARβ/δ could represents an important function of the receptor. To provide evidence of the involvement of PPARβ/δ in the induction of VEGF we knocked it down using RNA interference. The efficiency of the siRNA-mediated knock-down was confirmed by RT-PCR (Fig. 5A). PPARβ/δ knock-down reduced the basal level of VEGF mRNA and the ability of GW501516 to induce VEGF mRNA compared to control transfected cells (Fig. 5A). The effect of PPARβ/δ depletion was more evident in H358 than H441 cells, probably because of the lower initial level of the receptor. Similar results were obtained with the direct PPARβ/δ target ADRP. Basal ADRP expression and the response to GW501516 was lower in PPARβ/δ depleted cells compared to control transfected cells (Fig. 5A). Interestingly, knock-down of PPARβ/δ slightly reduced the basal level of VEGFR1 and VEGFR2 and enhanced, rather than antagonize, the effect of GW501516 (Fig. 5A). Thus, depletion of PPARβ/δ had opposite effects on VEGF and VEGFRs, consistent with the notion that PPARs can affect gene expression by distinct mechanisms [26].

In addition to siRNA-mediated knock-down, we tested the PPARβ/δ antagonist GSK0660 [27]. Cells were incubated for 2 h with GSK0660 (10 µM) prior to treatment with GW501516. This dose of GSK0660 did not affect cell viability but effectively inhibited PPARβ/δ activity. GSK0660 had limited effects on the basal level of VEGF mRNA, but blocked the induction of VEGF by GW501516 (Fig. 5A). GSK0660 had a similar effect on ADRP mRNA both in basal condition and upon ligand activation. GSK0660 reduced also the level of VEGFR1 and VEGFR2 mRNA compared to control cells both in basal conditions and in the presence of GW501516 (Fig. 5B). Thus, both siRNA mediated knock-down and the PPARβ/δ antagonist blocked VEGF transcription, consistent with the hypothesis that PPARβ/δ was directly involved in the process and acted as a transcriptional activator.

To determine whether PPARβ/δ regulated VEGF expression by direct binding to the VEGF promoter, we performed chromatin immunoprecipitation and assessed binding of PPARβ/δ to a region of the VEGF promoter (−527/−298) containing a PPRE [28]. An upstream region of the VEGF promoter (−1338/−1123) lacking PPREs was used as negative control. Upon ligand activation, binding of PPARβ/δ was detected to the PPRE containing site while no binding was detected in the distal region (Fig. 5C). Densitometric analysis of the results confirmed binding of PPARβ/δ to the VEGF promoter upon ligand activation, while there was no binding in the absence of ligand and in IgG control samples (Fig. 5C). Furthermore, the extent of PPARβ/δ binding to the VEGF promoter was comparable to that to ADRP promoter, a known of PPARβ/δ target (Fig. 5C).

### PI3K Activation is Involved in VEGF Induction by PPARβ/δ

**Figure 6:**
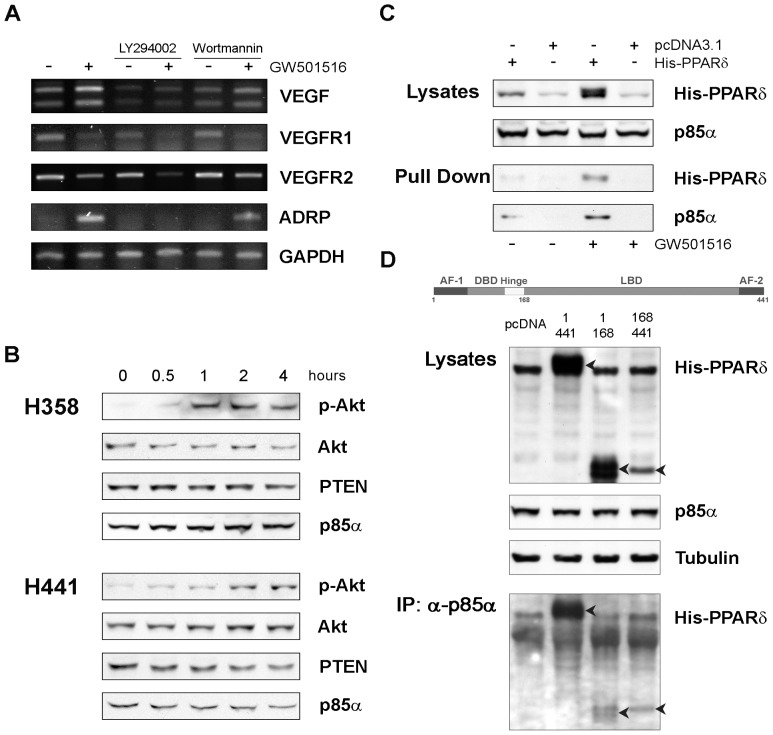
PI3K/Akt activation by PPARβ/δ agonists. (**A**) H358 cells were grown to confluence, starved for 24 hours and then incubated for 2 h with LY294002 (25 µM) or wortmannin (200 nM) followed by GW501516 (5 µM) for 18 h. RNA was extracted and analyzed by RT-PCR. (**B**) H358 and H441 cells were incubated with and without GW501516 for the indicated time. Cell lysates were examined by immunoblotting with antibodies for total and phosphorylated Akt, PTEN and p85α. (**C**) H358 cells were transfected with His-PPARβ/δ or empty pcDNA3.1 vector and incubated with and without GW501516 for 18 h. Cells were lysed in RIPA buffer and His-PPARβ/δ was pulled down with His-select nickel affinity gel. Immunoblots were developed with antibodies for PPARβ/δ and p85α. (**D**) H358 cells were transfected with pcDNA3.1, full length His-PPARβ/δ (PPARβ/δ 1–441), or truncated PPARβ/δ retaining the N-terminal part (PPARβ/δ 1–168) and C-terminal part (PPARβ/δ 168–441), lysed in RIPA buffer and subject to immunoprecipitation with anti-p85α antibody. Immunoblots of whole cell lysates and immunoprecipitates (*middle and bottom panel*, respectively) were performed with antibodies directed to p85α, tubulin and His-tag. Arrows indicate full length and truncated PPARβ/δ detected in immunoblots of whole cell lysates and immunoprecipitates. *Top panel*, schematic representation of PPARβ/δ structure and domain organization.

We explored whether additional pathways that have been associated to PPARβ/δ could contribute to the regulation of VEGF in response to PPARβ/δ agonists. The PI3K/Akt pathway is activated in many cancers and is implicated in tumor angiogenesis [29]. ILK and PDK, two downstream target kinases of Akt, were shown to be regulated by PPARβ/δ [24] and activation of PPARβ/δ increased phosphorylated Akt (pAkt) in endothelial progenitor cells [30] and NSCLC cells [31]. Therefore, we examined whether the PI3K/Akt pathway was involved in VEGF induction by PPARβ/δ agonists in NSCLC cells. Cells were pretreated for 2 h with the PI3K inhibitor LY294002 followed by GW501516. Pre-incubation with LY294002 at doses known to inhibit Akt phopshorylation reduced the expression of VEGF and prevented the induction of VEGF in response to the PPARβ/δ ligand (Fig. 6A). LY294002 had a similar effect on ADRP. However, the PI3K inhibitor did not block down-regulation of VEGFR1 and VEGFR2 induced by GW501516. Wortmannin, another potent PI3K inhibitor, had similar effects on VEGF, ADRP and VEGFRs (Fig. 6A).

Together, these data indicated that PI3K contributed to PPARβ/δ mediated induction of VEGF. The phosphatase and tensin homolog deleted on chromosome 10 (PTEN) is a major regulator of PI3K activity [29]. Modulation of the PI3K/Akt pathway in response to activation of PPARs has been attributed to changes in PTEN level. PPARγ agonists increased PTEN with consequent inhibition of PI3K [32]. On the other hand, activation of PPARβ/δ reduced PTEN leading to increased pAkt [22], [31]. However, when we treated H441 and H358 cells with GW501516 we did not observe any consistent change in PTEN level (Fig. 6B). Moreover, we found that GW501516 induced pAkt at very early time. Increased pAkt was seen within 1–2 h of treatment and was sustained for at least 4 h (Fig. 6B). Total Akt, PTEN and p85α were not or minimally affected at this time, suggesting that changes in the level of these proteins were unlikely to account for the induction of pAkt by the PPARβ/δ agonist.

Several studies have shown that PI3K can be activated by physical interaction of the PI3K regulatory subunit p85α with NHRs. Therefore, we assessed whether PPARβ/δ was able to interact with p85α in NSCLC cells. p85α co-precipitated with His-PPARβ/δ in pull-down experiments and the interaction was enhanced by the presence of GW501516 (Fig. 6C). p85α was not detected in His-PPARβ/δ pulldown in control transfected cells with and without GW501516. We examined the binding of full length and truncated forms of PPARβ/δ to p85α also by immunoprecipitation with anti-p85α antibody to determine whether the interaction involved specific portions of the receptor. His-tagged truncated forms of PPARβ/δ, which retained the N-terminal portion (PPARβ/δ 1–168; including the AF1, DNA Binding Domain and hinge region) or the C-terminal portion (PPARβ/δ 168–441; including the Ligand Binding Domain and AF2) interacted with p85α similar to full length PPARβ/δ (Fig. 6D). However, when we normalized to the level of expression, the C-terminal portion seemed to contribute more than the N-terminal portion to the binding of p85α. Together, these data demonstrated direct binding of PPARβ/δ to p85α and provide a mechanistic explanation for the induction pAkt by PPARβ/δ agonists.

## Discussion

PPARβ/δ is over-expressed in many human cancers and has been shown to promote proliferation and survival in various cancer cell types and tumor models [1]. Altogether, these findings suggested that PPARβ/δ might have a tumor promoting function, although this is not universally accepted [9]. In this study, we investigated the consequences of PPARβ/δ activation and the involvement of the receptor in various metabolic and signaling pathways in NSCLC cells. The role played by PPARβ/δ in NSCLC has been investigated previously [22], [31], [33]. However, the results obtained in different experimental models were controversial [23]. We found that PPARβ/δ agonists promote proliferation and survival of NSCLC cells, while knock-down of PPARβ/δ reduces proliferation and increases cell death. An important factor in determining the response to PPARβ/δ agonists was the cellular level of the receptor. Cells with relatively high PPARβ/δ activity (e.g., H441 and H358) exhibited a proliferative and pro-survival response, which was absent or minimal in cells with low level of the receptor (e.g., A549). Additional factors present in the cells, such as the level of Cox-2, PGES, cPLA_2_, PPARγ and endogenous ligands, might also influence the type of response to PPARβ/δ agonists. Similar paradoxical effects were described with all-*trans* retinoic acid (RA), which was found to bind to PPARβ/δ in addition to the retinoic acid receptor (RAR) [34]. The type of pro- or anti-apoptotic response elicited by RA depended on the relative level of RAR and PPARβ/δ and the presence of specific cofactors that drive activation of either receptor. Notably, consistent with our data, RA signaling through PPARβ/δ had potent anti-apoptotic and tumor promoting effects.

Our data indicate that PPARβ/δ regulates important factors in tumorigenesis, like VEGF and Cox-2. Both Cox-2 and VEGF had been previously identified as targets of PPARβ/δ [18], [35]. We observed that both VEGF and Cox-2 were induced by PPARβ/δ agonists in NSCLC cells. Furthermore, we found increased expression of PPARβ/δ, Cox-2 and VEGF in a large fraction of NSCLC samples compared to normal lung. Interestingly, a recent study found that concomitant over-expression of PPARβ/δ and Cox-2 in colorectal cancers was associated with reduced patient survival [36]. Notably, we found that PPARβ/δ regulated VEGF in NSCLC cells through a dual mechanism. In addition to ligand-induced binding of PPARβ/δ to the VEGF promoter, the induction of VEGF depended on the activation of PI3K through a non-genomic mechanism that was blocked by PI3K inhibitors. We found that PPARβ/δ and p85α physically interacted and that PPARβ/δ agonists enhanced the interaction leading to activation of PI3K and phosphorylation of Akt. The requirement for PPARβ/δ agonists for induction of pAkt suggested that the receptor might associate with protein cofactors or undergo a conformational change in order to promote the activation of the PI3K catalytic subunit. Various NHRs, including PPARβ/δ, were reported to interact with p85α and activate PI3K/Akt making this a relevant route of activation of this key signaling pathway [30], [37], [38], [39]. Furthermore, cross-talks between the PI3K/Akt and Cox-2/prostaglandin synthetic pathway, which might be highly relevant for tumor progression, might be mediated through PPARβ/δ. Interestingly, while increasing VEGF transcription, activation of PPARβ/δ led to a decrease of VEGFR1 and VEGFR2 mRNA, which are the major mediator of VEGF function [40]. This suggest that VEGF might exert paracrine rather than autocrine functions in NSCLC cells favoring the effects on surrounding endothelial and stromal cells.

PPARβ/δ appears to be a central node of multiple signaling pathways, being able to regulate various processes including proliferation, survival, inflammation, angiogenesis and cell metabolism. PPARβ/δ might function as a sensor of metabolic and inflammatory states in the tumor microenvironment and, in this context, activate pro-survival, pro-inflammatory and pro-angiogenic responses in tumor cells and the surrounding stroma. This would be consistent with the concomitant up-regulation of PPARβ/δ along with Cox-2, VEGF, cPLA_2_ and PGES often seen in NSCLC. cPLA_2_/Cox-2/PGES might form feed-forward loops with PPARβ/δ sustaining a pro-tumorigenic response by stimulating cell survival, inflammation and angiogenesis. PGE_2_ produced by the action of Cox-2 and PGES, although it is not a direct agonist, can indirectly contribute to activation of PPARβ/δ, which in turn would promote Cox-2 expression, prostaglandin synthesis and VEGF production. On the other hand, it seems unlikely that PGIS has a pro-tumorigenic role in NSCLC. In this study we found unchanged or decreased expression of PGIS in NSCLC compared to normal lung. Furthermore, PGIS was not or minimally expressed in NSCLC cell lines. A previous study also did not find changes of PGIS expression in lung tumors, although both Cox-2 and PGES were up-regulated [16]. These findings, therefore, are in line with a protective role of PGIS and PGI_2_ against lung carcinogenesis. They are also consistent with recent studies with the synthetic PGI_2_ analogue iloprost and PGIS transgenic mice [41]. The tumor suppressive effects of PGI_2_ might in fact be independent of PPARβ/δ and related to activation of PPARγ as seen with synthetic analogues [42].

NSCLC has a very poor prognosis and very few drugs are effective in prolonging survival of lung cancer patients. Understanding the biology of NSCLC is therefore of primary importance to develop new therapeutic approaches. In this study, we show that PPARβ/δ controls multiple metabolic and signaling pathways contributing to various aspects of lung cancer. Strategies to interfere with PPARβ/δ might therefore be beneficial as they might concomitantly affect multiple critical pathways involved in lung tumorigenesis.

## Supporting Information

Figure S1 Expression of PPARγ, cPLA_2_, Cox-2, VEGF, PGIS, and PGES in non-small cell lung cancers and adjacent normal tissue.
(PDF)

Figure S2 Effects of ciglitazone, sulindac sulfide, sulindac sulfone, and NS398 on growth of H441 and A549 cells.
(PDF)

Table S1 Sequences of PCR primers and siRNAs.
(PDF)

Table S2 Correlation analysis of PPARβ/δ, VEGF and Cox-2 expression in human lung cancer microarray datasets.
(PDF)
