# Supplementary material for: Transcriptional and Non-Transcriptional Functions of PPARβ/δ in Non-Small Cell Lung Cancer
Source: PLoS One. 2012 Sep 25;7(9):e46009. doi: 10.1371/journal.pone.0046009 (PMC3457940; doi:10.1371/journal.pone.0046009)
Supplement: Figure S1 — Expression of PPARγ, cPLA2, Cox-2, VEGF, PGIS, and PGES in non-small cell lung cancers and adjacent normal tissue. (PDF) [file pone.0046009.s001.pdf]

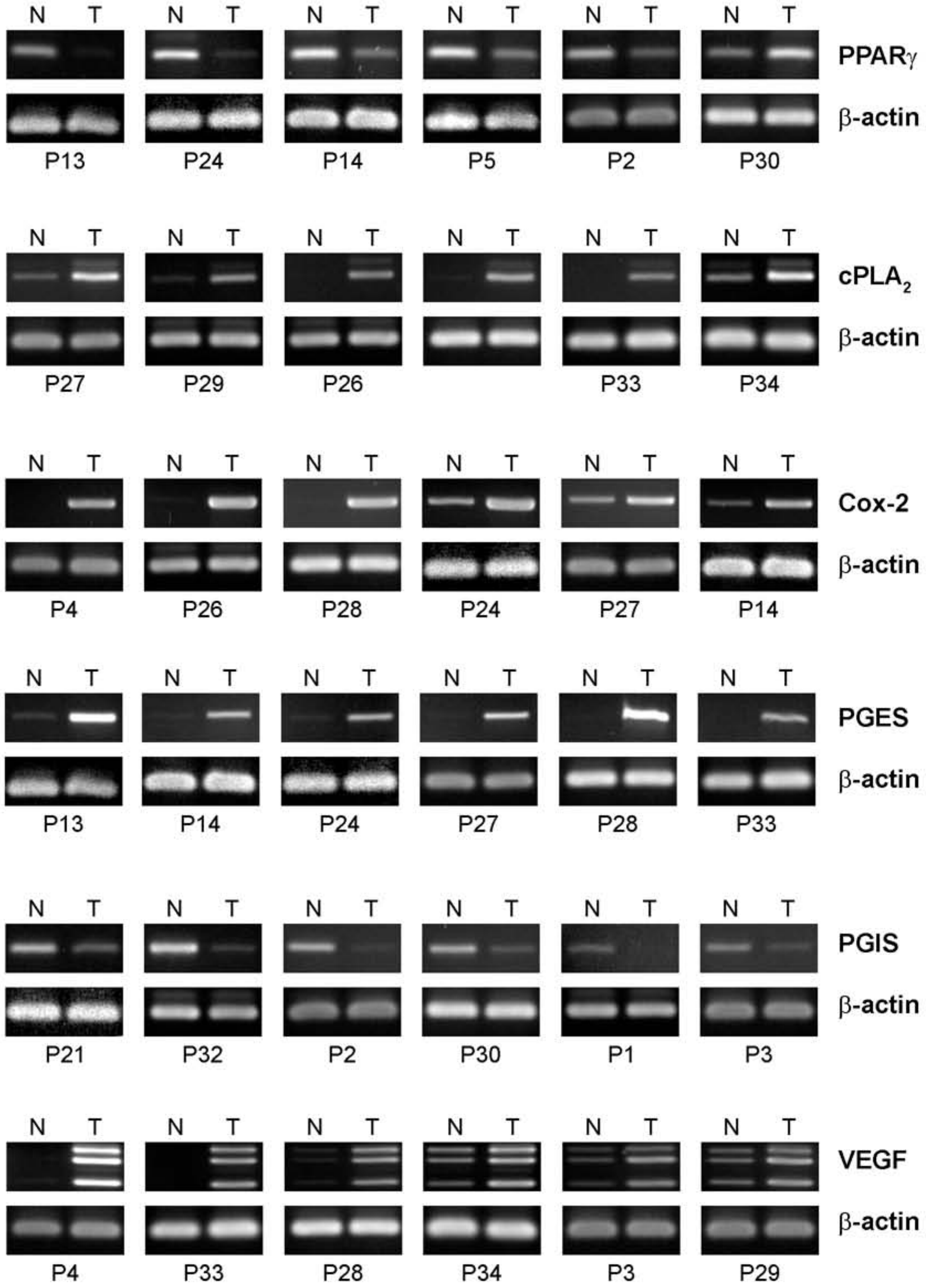

Figure S1. Expression of PPAR, cPLA2, Cox-2, VEGF, PGIS, and PGES in non-small cell lung cancers and adjacent normal tissue. RNA was extracted from tumors and adjacent normal lung tissue from patients with non-small cell lung cancer and examined by RT-PCR with gene specific primers. -actin was used as reference gene. Representative gel images are shown.
