# Supplementary material for: Transcriptional and Non-Transcriptional Functions of PPARβ/δ in Non-Small Cell Lung Cancer
Source: PLoS One. 2012 Sep 25;7(9):e46009. doi: 10.1371/journal.pone.0046009 (PMC3457940; doi:10.1371/journal.pone.0046009)
Supplement: Figure S2 — Effects of ciglitazone, sulindac sulfide, sulindac sulfone, and NS398 on growth of H441 and A549 cells. (PDF) [file pone.0046009.s002.pdf]

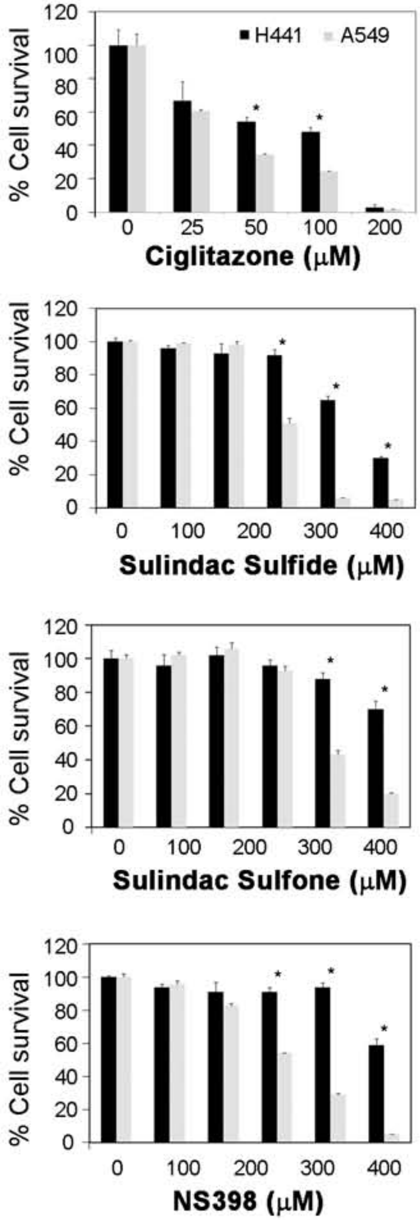


Figure S2. Effects of ciglitazone, sulindac sulfide, sulindac sulfone, and NS398 on growth of H441 and A549 cells. H441 and A549 cells were plated in 96-well plates and after 24 h incubated with the indicated compounds. Cell viability was assessed after 72 h with MTT assay. *P<0.01
