# Supplementary material for: Transcriptional and Non-Transcriptional Functions of PPARβ/δ in Non-Small Cell Lung Cancer
Source: PLoS One. 2012 Sep 25;7(9):e46009. doi: 10.1371/journal.pone.0046009 (PMC3457940; doi:10.1371/journal.pone.0046009)
Supplement: Table S1 — Sequences of PCR primers and siRNAs. (PDF) [file pone.0046009.s003.pdf]

Table S1. Sequences of PCR primers and siRNAs

Gene		Sequence
**Primers (RT-PCR)**		
β-Actin	Forward	5′-AAGAGAGGCATCCTCACCCT-3′
	Reverse	5′-TACATGGCTGGGGTGTTGAA-3′
ADRP	Forward	5′-CCTTGGTGAGCTCCACGTAT-3′
	Reverse	5′-CTTCTCCACACTGCCAGTCA-3′
Cox-2	Forward	5′-TGTGGGGCAGGAGGTCTTGGTCT-3′
	Reverse	5′-GCATCTGGCCGAGGCTTTTCTAC-3′
$cPLA_2$	Forward	5′-CCAAAGTGACAAAGGGGGCC-3′
	Reverse	5′-GCTACCACAGGCACATCACG-3′
GAPDH	Forward	5′- GGCTGTGGGCAAGGTCATCCCTGA-3′
	Reverse	5′- TCCACCACCCTGTTGCTGTA-3′
ILK	Forward	5’-GTACCTGACCTGCCCACACT-3’
	Reverse	5’- TCTCGAACCTTCAGCACCTTCACGACAA -3’
PDK	Forward	5′-TTCGTCCTCCTCCTCACACT-3′
	Reverse	5′-GCCTCTGGTCGAAGTTCTTG-3′
PPARδ	Forward	5’-GGCCTTCTCCAAGCACATCTA-3’
	Reverse	5’-TGCGCAGGAACTCACGGGTGA-3’
VEGF	Forward	5′-ATGAACTTTCTGCTGTCTTGGGTGCATT-3′
	Reverse	5′-TCACCGCCTCGGCTTGTCACAT-3′
VEGFR1	Forward	5′-CAAGTGGCCAGAGGCATGGAGTT-3′
	Reverse	5′-CTGTGCCAGCAGTCCAGCAT-3′
VEGFR2	Forward	5′-GAGGGCCACTCATGGTGATTGT-3′
	Reverse	5′-CGTGCCAGCAGTCCAGCAT-3′
**Primers (ChIP)**		
-527/-298	Forward	5’-AGAGTGAGGACGTGTGTGTCTGT-3’
	Reverse	5’-GAGCAGGAAAGTGAGGTTACGTG-3’
-1338/-1123	Forward	5’-AAGGATAGGGGAGAAGCTGTGAG-3’
	Reverse	5’-CTGCGTGATGATTCAAACCTACC-3’
**siRNA**		
PPARδ	Forward	5’-GGCCUUCUCCAAGCACAUCtt-3’
	Reverse	5’-GAUGUGCUUGGAGAAGGCCtt-3’
GL3	Forward	5’- CUUACGCUGAGUACUUCGAtt -3’
	Reverse	5’- UCGAAGUACUCAGCGUAAGtt -3’
