# Supplementary material for: Transcriptional and Non-Transcriptional Functions of PPARβ/δ in Non-Small Cell Lung Cancer
Source: PLoS One. 2012 Sep 25;7(9):e46009. doi: 10.1371/journal.pone.0046009 (PMC3457940; doi:10.1371/journal.pone.0046009)
Supplement: Table S2 — Correlation analysis of PPARβ/δ, VEGF and Cox-2 expression in human lung cancer microarray datasets. (PDF) [file pone.0046009.s004.pdf]

Table S2. Correlation analysis of PPARβ/δ, VEGF and Cox-2 expression in human lung cancer microarray datasets

Lung Cancer Dataset	Number of samples	Gene	Pearson R	Pearson p-value
**Rohrbeck**	47	VEGFA	0.59	1.3E-05
		Cox-2	0.50	3.7E-04
**Garber**	73	VEGFA	0.44	1.1E-04
		Cox-2	nc	ns
**Hou**	156	VEGFA	0.26	0.001
		Cox-2	0.25	0.001
**DirectorsChallenge**	443	VEGFA	0.25	8.7E-08
		Cox-2	nc	ns

Genome-wide transcriptome datasets from four independent studies (PMID: 18992152, 11707590, 20421987, 18641660) including human lung cancer and normal lung samples were downloaded and normalized gene expression values for PPARβ/δ, VEGF and Cox-2 were used for correlation analysis. Pearson correlation coefficient and corresponding p-values of VEGFA and COX-2 expression with respect to PPARβ/δ were calculated for the samples in each dataset. *nc*, no correlation. *ns*, non significant.
